# Quantification of Corn Adulteration in Wet and Dry-Processed Peaberry Ground Roasted Coffees by UV–Vis Spectroscopy and Chemometrics

**DOI:** 10.3390/molecules26206091

**Published:** 2021-10-09

**Authors:** Meinilwita Yulia, Diding Suhandy

**Affiliations:** 1 Department of Agricultural Technology, Lampung State Polytechnic, Jl. Soekarno Hatta No. 10, Rajabasa, Bandar Lampung 35141, Indonesia; meinilwitayulia@polinela.ac.id; 2 Department of Agricultural Engineering, Faculty of Agriculture, The University of Lampung, Jl. Soemantri Brojonegoro No.1, Bandar Lampung 35145, Indonesia

**Keywords:** UV–Vis spectroscopy, peaberry coffee, individual model, global model, dry bean processing, wet bean processing, adulteration, authentication, partial least squares regression, multiple linear regression

## Abstract

In this present research, a spectroscopic method based on UV–Vis spectroscopy is utilized to quantify the level of corn adulteration in peaberry ground roasted coffee by chemometrics. Peaberry coffee with two types of bean processing of wet and dry-processed methods was used and intentionally adulterated by corn with a 10–50% level of adulteration. UV–Vis spectral data are obtained for aqueous samples in the range between 250 and 400 nm with a 1 nm interval. Three multivariate regression methods, including partial least squares regression (PLSR), multiple linear regression (MLR), and principal component regression (PCR), are used to predict the level of corn adulteration. The result shows that all individual regression models using individual wet and dry samples are better than that of global regression models using combined wet and dry samples. The best calibration model for individual wet and dry and combined samples is obtained for the PLSR model with a coefficient of determination in the range of 0.83–0.93 and RMSE below 6% (*w*/*w*) for calibration and validation. However, the error prediction in terms of RMSEP and bias were highly increased when the individual regression model was used to predict the level of corn adulteration with differences in the bean processing method. The obtained results demonstrate that the use of the global PLSR model is better in predicting the level of corn adulteration. The error prediction for this global model is acceptable with low RMSEP and bias for both individual and combined prediction samples. The obtained RPD_p_ and RER_p_ in prediction for the global PLSR model are more than two and five for individual and combined samples, respectively. The proposed method using UV–Vis spectroscopy with a global PLSR model can be applied to quantify the level of corn adulteration in peaberry ground roasted coffee with different bean processing methods.

## 1. Introduction

Specialty coffee is a premium product and, according to the Specialty Coffee Association of Europe [1], “Specialty coffee is defined as a crafted quality coffee-based beverage, which is judged by the consumer (in a limited marketplace at a given time) to have a unique quality, a distinct taste and personality different from, and superior to, the common coffee beverages offered. The beverage is based on beans that have been grown in an accurately defined area, and which meet the highest standards for green coffee and its roasting, storage, and brewing”. In Indonesia, specialty coffee can be *Coffea liberica*, *Coffea arabica,* or *Coffea canephora*. In the market, three types of commercially traded specialty coffee are available: single-origin coffees (including Gayo coffee, Kalosi coffee, Mandailing coffee, Toraja coffee, and Lampung coffee), digested animal coffees (including wild civet coffee, feeding civet coffee, and bat coffee), and peaberry coffee (a single bean/monocotyledon) [2,3].

Nowadays, the growth of specialty coffee consumption is faster than that of the traditional one [2]. Mostly driven by economic motivation, food fraud, both in terms of mislabeling and adulteration, is now increasing and becoming a serious problem in specialty coffee trading. For example, it was reported that 42% of commercial civet coffee was fake or adulterated with normal non-civet coffee [4]. For peaberry specialty coffee, the adulteration frequently happens in the form of ground roasted coffee, since after roasting and grinding, the discrimination of ground coffee made from peaberry and traditional (normal) coffee is almost impossible with the conventional methods [5,6]. For this reason, several sensitive emerging analytical methods to quantify adulterants in coffee have been developed in the past ten years: high-performance liquid chromatography (HPLC) [7], gas chromatography-mass spectrometry (GC-MS) [8], electrospray ionization mass spectrometry (ESI-MS) [9], and real-time polymerase chain reaction (RT-PCR) [10]. However, these accurate methods are expensive in instrumentation and require a highly trained person. 

The spectroscopic-based method, using different electromagnetic regions along with chemometrics, has been successfully applied for cereal adulteration quantification in ground roasted coffee, both in single and multiple adulterants using near-infrared (NIR), ultraviolet–visible (UV–Vis), mid-infrared, Raman, nuclear magnetic resonance (NMR), and laser-induced breakdown spectroscopy (LIBS) [11,12,13,14,15,16]. Most of these methods are less expensive in the device and faster in sample preparation (little or no need for sample preparation). Some previous works have incorporated the variation of postharvest treatments in coffee samples such as differences in coffee roasting (light, medium, and dark) in the developed calibration model [17,18]. However, in the aforementioned studies, no reported works included the influence of other important postharvest factors especially the bean processing method in the developed calibration models. Previously, Suhandy and Yulia [19] showed a significant influence of differences in the bean processing method (dry, wet, and semi-dry) on the discrimination of Lampung robusta specialty ground roasted coffee. For green bean coffee, Barrios-Rodríguez et al. [20] successfully demonstrated the significant discrimination between the wet, dry, and semi-dry processing method of *Coffea arabica* L. var. Colombia using infrared spectroscopy coupled with chemometrics. 

In this study, corn was selected as an adulterant material due to its low cost and huge availability in the Indonesian market. Additionally, corn is one of the most used diluents in coffee adulteration as reported in several previous works [15,16,21,22,23]. Therefore, in this present research, we evaluate a spectroscopic method based on UV–Vis spectroscopy and chemometrics to quantify the corn adulteration in coffee involving two common types of bean processing of wet and dry-processed methods. More specifically, the objective of this study is to investigate a robust calibration model using three different linear regression methods, including PLSR, MLR, and PCR, for the quantification of corn adulteration in peaberry specialty coffee incorporated with different bean processing methods.

## 2. Materials and Methods

### 2.1. Peaberry Samples and Their Adulteration

Green bean peaberry coffee samples with two types of bean processing method (wet and dry with about 1 kg each) were obtained from a certified coffee supplier located in Garut, West Java province, Indonesia. The peaberry green bean samples are specialty grade from mixed cultivars of *Coffea arabica* L. and its hybrid (mostly Sigarar utang, Lini S, Ateng super, Catimor, and Typica) harvested in the year 2019 and originating from Cikuray, Papandayan, and Kamojang mountainous coffee plantation in Garut, West Java province, Indonesia (latitude and longitude coordinates 7°19′22.4″ S and 107°51′37.9″ E, respectively; altitude, ±1600 m). 

Before roasting by portable roaster (at 200 °C for 15 min), all beans were visually inspected and showed no defective grains. After roasting, the unroasted and over-roasted beans were removed carefully by hand. After grinding, particles of size 40 mesh (400 µm) were obtained, which were used to perform all physicochemical analyzes.

Corn with its low cost and huge availability in the Indonesian market was selected as an adulterant. Corn samples were collected from a local farmer in Lampung province, Indonesia. According to Sezer et al. [16], with modification, corn was roasted in two steps: at 100 °C for 7 min and followed by 200 °C for 10 min, ground (Sayota home grinder), and mechanically sieved through a U.S. mesh size 40 to obtain the same particle size for all the samples (400 µm). The wet and dry-processed peaberry ground roasted coffees were intentionally adulterated with the ground roasted corn in the range of 10–50% (*w*/*w*) with an increment of 10% (*w*/*w*). This adulteration range was chosen according to several previous works [15,16]. It is also the most common adulteration level found in the Indonesian markets [12].

**Figure 1:**
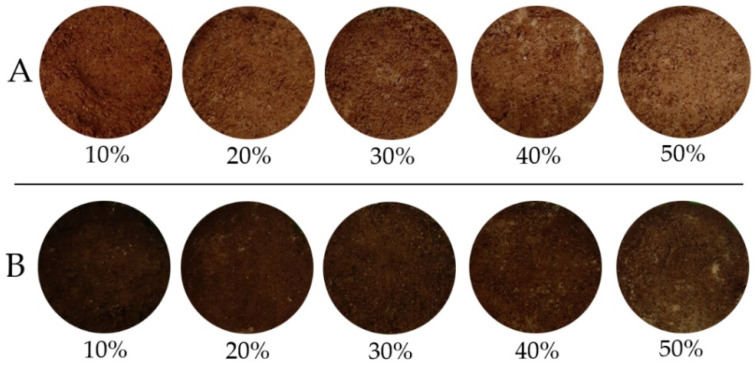
The visual appearance of peaberry dry-processed (**A**) and wet-processed (**B**) coffee with 10–50% of corn adulteration.

Total 199 samples (1 g each) of adulterated peaberry dry and wet-processed coffees were provided. They consisted of 20 samples for each level of corn adulteration, resulting in a total of 100 samples for dry-processed peaberry coffees and 99 samples for wet-processed peaberry coffees (19 samples were provided at a level of 40% for wet-processed peaberry coffees). Figure 1 shows its visual appearance with 10–50% of corn adulteration level before extraction with hot distilled water. The adulterated peaberry wet-processed samples were darker than that of peaberry dry ones. However, it was visually difficult to discriminate between the different levels of adulteration in both wet and dry adulterated peaberry samples. 

### 2.2. Sample Extraction and UV–Vis Spectral Data Measurement

Coffee samples were extracted based on a standard procedure as reported by previous studies [5,12]. Raw UV–Vis spectral data was obtained for aqueous samples in the range between 250 and 400 nm with 1 nm interval using a UV–visible spectrometer (Genesys™ 10S UV–Vis, Thermo Scientific, Waltham, MA, USA) in .csv format. After reformatting into .xls, the raw spectral data were imported to the Unscrambler X ver. 10.4 (CAMO Software AS, Oslo, Norway) for chemometrics analysis.

### 2.3. Chemometrics

Since there is no standard protocol for spectral pre-processing, a trial and error approach was adopted. Different spectral pre-preprocessing was available in the Unscrambler X ver. 10.4 (CAMO Software AS, Oslo, Norway) to reduce or to remove the effect of several different unwanted interfering phenomena such as particle size influence (baseline different and light scattering), etc. As mentioned by Roger et al. [24] and Bian et al. [25], it is hard to determine which pre-processing can successfully improve the given original spectral data. For this reason, instead of selecting the best pre-processing, to optimize the effect of spectral pre-processing, the combination of several spectral pre-processing methods is often used [19]. To eliminate noise and systematic spectra variation, three consecutive spectral pre-processing methods were found to be the best applied: moving averaging smoothing with 5 segments (MAS), standard normal variate (SNV), and Savitzky–Golay first derivative with 11 smoothing gaps and second-order polynomial (SG1d). MAS was widely used to smooth the spectral data before applying various pre-processing methods [26]. SNV was effective in normalizing the spectra for canceling the scattering effect, while SG1d was used to correct the baseline effect [27,28]. Due to similarity in coffee species of both samples of wet and dry-processed coffees, it was expected that the spectral difference in peaberry coffee samples due to differences in the level of adulteration between wet and dry-processed coffees was small. The SG1d spectral pre-processing was also used to enhance these small spectral differences [26]. 

PCA (principal component analysis), which is widely used in analytical chemistry [18], was used to study any possible clustering of adulterated peaberry samples according to the differences in bean processing methods. The plot of the score and its corresponding x-loadings from the first two principal components (PCs) was presented for raw and preprocessed spectra.

Among numerous multivariate linear regression methods for quantification of adulteration in coffee, the partial least squares regression (PLSR) is widely used. In this research, we applied PLSR and compared it to other linear methods of multiple linear regression (MLR) and principal component regression (PCR) to quantify the level of corn adulteration. PLSR and PCR were developed using spectral data from 250 to 400 nm (number of variables = 161). In MLR, a selected few variables were obtained from a plot of x-loadings. Wavelengths that were associated with the positive and negative peaks were used as input. All regression models were validated by the full cross-validation method to optimize the model parameters.

According to Costa et al. [29] and Macedo et al. [30], the samples were manually selected and separated into two sets: a calibration and prediction set as presented in Table 1. The procedure of this separation of the samples was as follows: order the samples concerning the corn adulteration level (from minimum to maximum values); then, four samples were selected every five samples for the calibration and the rest for prediction. By doing this, as seen in Table 1, a more uniform selection of the calibration and prediction sample sets could be obtained. 

Table 2 shows the statistical parameters used to assess the quality of the calibration model and evaluate the performance of its prediction [31,32]. For model evaluation, the following statistical parameters were used, including the coefficient of determination of calibration and cross-validation (R^2^_c_ and R^2^_cv_), root means squared errors of calibration, and cross-validation (RMSEC and RMSECV), and the ratio of prediction to deviation in cross-validation (RPD_cv_). Limit of detection (LOD) and limit of quantification (LOQ) were also calculated according to Milani et al. [15] and Rambla-Alegre et al. [33].

In the prediction step, the performance of the regression model was evaluated using the following statistical parameters: the coefficient of determination for prediction (R^2^_p_), standard error of prediction (SEP), bias, root mean square error of prediction (RMSEP), RPD, and RER in prediction. The RPD is the ratio of the standard deviation of reference data for the validation or prediction set to RMSECV or RMSEP and the RER is the ratio between the difference of the maximum and minimum reference values for the data in the prediction set to RMSEP [34]. 

### 2.4. Software

Chemometrics and spectral analysis were calculated using the Unscrambler X ver. 10.4 (CAMO Software AS, Oslo, Norway).

## 3. Results and Discussion

### 3.1. Spectral Data of Wet and Dry Peaberry Coffees with Different Levels of Corn Adulteration

**Figure 2:**
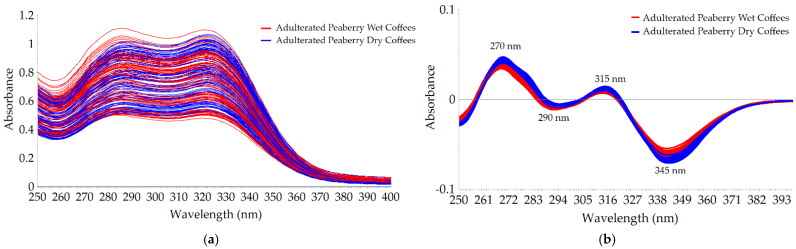
Spectral data of peaberry wet and dry-processed coffee with 10–50% of corn adulteration in the range between 250 and 400 nm: (**a**) raw spectra; (**b**) pre-processed spectra (MAS + SNV + SG1d).

Figure 2a shows the typical raw spectral data of adulterated peaberry wet and dry coffees in the range between 250 and 400 nm. Our spectra were similar to the work reported by Souto et al. [35]. The raw spectra were broad and overlapped; hence, it was hard to differentiate between the wet and dry adulterated peaberry. A better visualization was obtained using the preprocessed spectra as seen in Figure 2b. In general, the intensity of absorbance in dry adulterated peaberry coffees was higher than that of the wet ones, and it was in line with the previously reported work [19].

Several positive and negative peaks were observed clearly in the pre-processed spectral data (MAS + SNV + SG1d). The highest positive peak at 270 nm of pre-processed spectra was closely related to the C=O chromophore in caffeine molecules as reported by some previous works [35,36], indicating the significant difference of the caffeine content in adulterated wet and dry peaberry coffees. The negative peaks at 290 and 345 nm of pre-processed spectra corresponded with the absorbance of chlorogenic acids (CGA) of raw UV–Vis spectra in previous work [36]. Navarra et al. [37] reported a wavelength at 330 nm for the CGA absorbance when ethanol was used as the solvent. Dankowska et al. [36] reported a wavelength at 320 nm as one of the negative peaks found in the raw UV–Vis spectral data of arabica and robusta coffee and its adulteration using water as a solvent. In this study, with water used as the solvent, the peak of CGA of pre-processed spectral data was shifted to the longer wavelength at 345 nm. This shifting phenomenon was also found by the previous work by Souto et al. [35], with water used as the solvent, they found a wavelength shifting of CGA from 320 nm to 325 nm in raw UV–Vis spectral data.

### 3.2. PCA Scores and Loadings

**Figure 3:**
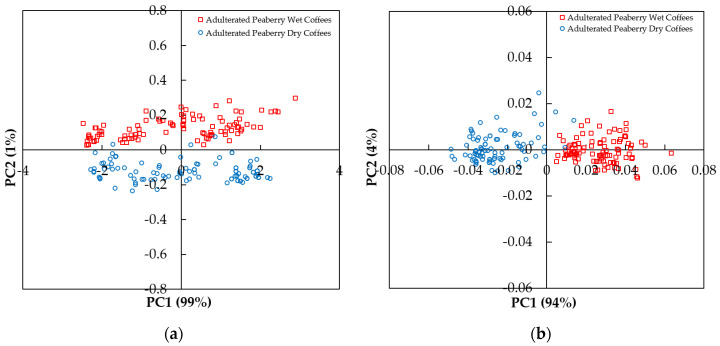
Plot of the first two principal components by PCA in the range between 250 and 400 nm: (**a**) raw spectra; (**b**) pre-processed spectra (MAS + SNV + SG1d).

**Figure 4:**
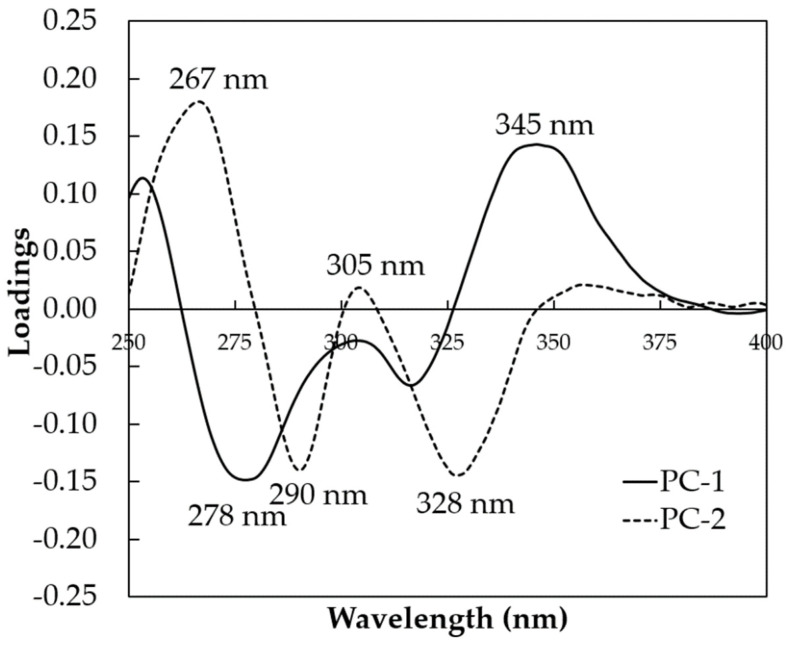
The plot of x-loading calculated by PCA in the range between 250 and 400 nm using pre-processed spectra.

Figure 3a shows the scores of the first two principal components (PC1 and PC2) of all coffee samples, including wet and dry with 10–50% of corn adulteration using raw spectral data in the range between 250 and 400 nm. The explained variance obtained for PC1 was high (PC1 = 99%). However, in terms of PC1, a good separation between the adulterated peaberry wet and dry coffees could not be achieved. A better PCA score plot was achieved using pre-processed spectral data in the range between 250 and 400 nm as presented in Figure 3b. Along PC1, with a 94% explained variance, all of the adulterated peaberry wet samples were plotted to the right of PC1 (PC1-positive), while most of the adulterated peaberry dry samples were on the left of PC1 (PC1-negative). Figure 4 shows the loadings plot of PC1 and PC2 using pre-processed spectral data. This plot shows the contribution of PC1 and PC2 to the separation of the adulterated peaberry wet and dry samples. In PC1 and PC2, the positive peaks with positive loading were observed at wavelengths of 267 and 345 nm. These wavelengths could be related to the absorbance of chlorogenic acids and trigonelline content in arabica coffee (CGA) [35], indicating that the adulterated peaberry wet sample coffees contained high contents of these compounds. This result was supported by previously reported work. Compared to the semi-dry method, Duarte et al. [38] reported that the wet coffees processed method showed higher contents of CGA and trigonelline due to a loss of other components with higher water solubility by lixiviation and thermal degradation during the wet processing. Three peaks with negative loadings were observed at wavelengths of 278, 290, and 328 nm. These wavelengths mainly contributed to discriminate against the adulterated peaberry dry coffees. Souto et al. [35] reported the maxima electronic absorption of trigonelline at 275 nm, caffeine at 280 nm, and caffeic acid at 325 nm using raw UV–Vis spectra. However, the adulterated peaberry dry coffees were mainly discriminated by the negative peak for PC1 at the wavelength of 278 nm, indicating that the adulterated peaberry dry sample coffees contained high contents of caffeine. It was supported by previous work [39]. It was reported that the caffeine content in dry processing coffees was higher, since about 40% of caffeine was removed with pulp during the wet processing [39]. These positive and negative peaks obtained from PCA x-loadings of pre-processed spectral data at 267, 278, 290, 305, 328, and 345 nm were used as input variables for constructing the MLR model.

### 3.3. Model Development for Quantification of Corn Adulteration

The correlation between pre-processed UV–Vis spectral data and the level of corn adulteration was quantified by developing three types of multivariate regression, including PLSR, MLR, and PCR using a calibration sample set and validated with a full-cross validation method. Three types of models were developed according to the range of samples: the individual wet model, individual dry model, and global model. For the individual wet and dry models, multivariate regression was developed using individual wet (*n* = 83) and dry (*n* = 84) calibration samples, respectively. For the global model, the multivariate regression was developed using a combined sample of wet and dry calibration samples (*n* = 167). The results are presented in Table 3. In general, all developed regression models had a sufficient and acceptable number of latent variables (LVs) ranging from four to nine. This met with the number of LVs not exceeding 15 as indicated by Bureau et al. [40]. A small difference between the RMSEC and RMSECV was also observed for the PLSR and PCR model, indicating the optimal number of LVs could be obtained [40]. The best individual wet model was obtained for the PLSR model using five LVs (explained 98% of the accumulated variance of the spectrum data and 92% of the score data) with R^2^_c_ = 0.93, R^2^_cv_ = 0.89, RMSEC = 3.85% (*w*/*w*), and RMSECV = 4.80% (*w*/*w*). For dry samples, the best individual dry model was also obtained for the PLSR model using six LVs (explained 97% of the accumulated variance of the spectrum data and 94% of the score data) with less accuracy than the individual wet model. However, both individual wet and dry models was acceptable with an RPD higher than two, indicating that PLS-DA models can be classified as excellent [41]. In a previous study, Sezer et al. [16] reported a similar result for the quantification of *Coffee arabica* adulteration with corn samples, employing laser-induced breakdown spectroscopy (LIBS) and PLS regression with R^2^_cal_ = 0.995, R^2^_val_ = 0.990, RMSEC = 4.32% (*w*/*w*), and RMSECV = 4.84% (*w*/*w*) being obtained. A better result was shown by Winkler-Moser et al. [17] for predicting corn adulteration using NIR spectroscopy with a lower error both in calibration and validation. They obtained a PLS model with R^2^_cal_ = 0.979, R^2^_val_ = 0.974, RMSEC = 1.05% (*w*/*w*), and RMSECV = 1.17% (*w*/*w*).

It was noted that, compared to the global model, all developed individual regression models using individual wet and dry samples had a better accuracy with a higher R^2^ and lower error count (both in terms of RMSEC and RMSECV). The PLS model was created to quantify both individual adulterant and global (combined) adulterants. The result showed that the PLS model for the quantification of individual adulterants was better than that of combined adulterants. According to Table 3, the PLSR model was superior compared to other regression models for the individual wet, dry, and global regression models. The RPD in cross-validation was more than two in all PLSR regression models (RPD critical = 2.0 [31,32]). According to Kapper et al. [42], all developed PLSR models showed a good accuracy with a high coefficient of determination between actual and predicted corn adulteration (R^2^ ≥ 0.70) both in calibration and validation.

**Figure 5:**
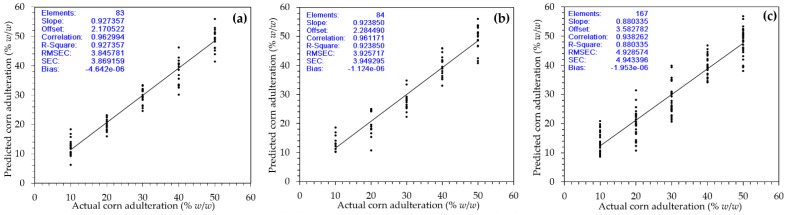
Actual versus predicted values of corn adulteration (% *w*/*w*) in peaberry coffee samples for the best PLSR model on (**a**) individual wet calibration samples, (**b**) individual dry calibration samples, and (**c**) combined calibration samples.

Figure 5 shows plots of the best PLSR calibration model for individual wet, dry, and combined calibration samples. Visually, it can be noticed that the residuals of calibration were randomly scattered closely to the regression line (bias was close to 0) for individual and combined calibration samples. The SEC and slope for individual calibration wet samples were 3.87% (*w*/*w*) and 0.93, resulting in an LOD and LOQ of 12.48% (*w*/*w*) and 41.61% (*w*/*w*), respectively. Similarly, the LOD and LOQ for individual calibration dry samples were 12.88% (*w*/*w*) and 42.93% (*w*/*w*). For combined calibration samples, the LOD and LOQ were 16.84% (*w*/*w*) and 56.14% (*w*/*w*). Compared to previous works, our result was inferior. For example, Milani et al. [15] reported satisfactory LOD values of 0.31–0.86% using the NMR spectroscopy with a different roasting profile. Sezer et al. [16] reported a quantitative approach using LIBS for coffee adulteration with different adulterants (corn, wheat, and chickpea) and resulting in a promising result with an LOD below 0.6% being obtained. The obtained LOD and LOQ using UV–Vis spectroscopy in this study were in the range of 12.48–16.84% and 41.61–56.14%. In this present study, a calibration and validation regression model was developed using corn adulterated samples in the range of 10–50% (*w*/*w*). However, the obtained LOD and LOQ in this study suggested we should have extended the range of corn adulteration up to more than 50%. For this reason, it needs an improvement for practical application. However, in Indonesia, the adulteration of more than 50% of specialty coffee is commonly found for economically motivated adulteration [12].

### 3.4. Prediction Using Individual and Global PLSR Models

To evaluate the influence of bean processing on the performance of the developed calibration model in prediction corn adulteration, the prediction was calculated on the individual (*n* = 16 for both individual wet and dry samples) and combined prediction sample sets (*n* = 32). The best individual and global PLSR models were used as the input. The results are presented in Table 4. The individual wet PLSR model resulted in a high RPD (RPD_p_ = 3.96) when it was used to predict corn adulteration in individual wet samples. However, this model failed to predict corn adulteration in individual dry samples resulting in low RPD_p_ (the RPD was less than one). The error prediction in terms of RMSEP and bias was highly increased. A similar result was found for prediction using the individual dry PLSR model. The individual dry PLSR model showed a good prediction with RPD_p_ = 3.33 for the prediction of dry samples and a failed prediction with RPD_p_ = 0.28 for the prediction of wet samples. The use of the global PLSR model was promising. The error prediction for this global model was acceptable with low RMSEP and bias for both individual and combined prediction samples. The RPD_p_ was higher than two for both individual predictions of wet and dry samples and combined samples. According to Chang et al. [43] and Valinger et al. [44] models with RPD > 2.0 are excellent descriptions and predictions of experimental data. In terms of RER, a global PLSR with RER in the range of 3 to 10, for both individual and combined prediction samples, is classified as a good practical utility model according to Jia et al. [45].

## 4. Conclusions

This research described the use of UV–Vis spectroscopy along with chemometrics to quantify the level of corn adulteration in peaberry specialty coffee with different bean processing methods. The proposed UV–Vis spectroscopy and global PLSR model detected an admixture of corn in the peaberry ground roasted coffee in the range of 10% to 50% with LOD values of 12.48–16.84% being reported for individual and combined samples. The reliability of the global PLSR model was confirmed by external validation using both individual (wet and dry) and combined prediction samples, indicating the great potential of UV–Vis spectroscopy and chemometrics as a green and low-cost analytical method for the authentication of peaberry specialty ground roasted coffee incorporated with different in bean processing methods.

## Figures and Tables

**Table 1:** Individual and global peaberry wet and dry-processed samples with 10–50% of corn adulteration in calibration and prediction sets. The range, mean, and standard deviations were expressed in % (*w*/*w*).

Individual Wet Samples	Calibration Set	Prediction Set
Number of samples	83	16
Range	10–50	10–50
Mean	29.88	30.00
Standard deviation (SD)	14.36	14.14
**Individual Dry Samples**		
Number of samples	84	16
Range	10–50	10–50
Mean	30.00	30.00
Standard deviation (SD)	14.31	14.14
**Global Samples**		
Number of samples	167	32
Range	10–50	10–50
Mean	29.94	30.00
Standard deviation (SD)	14.29	13.91

**Table 2:** Statistical parameters and their equations were used to assess the calibration model and its prediction performance.

Steps	Parameters	Equations ^1^	Accepted Values
Calibration	R^2^_c_ and R^2^_cv_	$1-\frac{\sum_{i=1}^{n}{\left(y_{i}-{\hat{y}}_{i}\right)}^{2}}{\sum_{i=1}^{n}{\left(y_{i}-{\bar{y}}_{i}\right)}^{2}}$	Close to 1
	RMSEC and RMSECV	$\sqrt{\frac{\sum_{i=1}^{n}{\left(y_{i}-{\hat{y}}_{i}\right)}^{2}}{n}}$	As low as possible
	RPD_cv_	$\frac{SD_{cv}}{RMSCV}$	More than 2
	LOD	$\frac{3\sigma }{S}$	As low as possible
	LOQ	$\frac{10\sigma }{S}$	As low as possible
Prediction	RMSEP	$\sqrt{\frac{\sum_{i=1}^{n}{\left(y_{i}-{\hat{y}}_{i}\right)}^{2}}{n}}$	As low as possible
	SEP	$\sqrt{{\left(RMSEP\right)}^{2}-{\left(bias\right)}^{2}}$	As low as possible
	bias	$\left(\overset{ˇ}{y}-\bar{y}\right)$	Close to 0
	RPD_p_	$\frac{SD_{pred}}{RMSEP}$	More than 2
	RER_p_	$\frac{y_{max}-y_{min}}{RMSEP}$	More than 10

^1^ n—number of samples; $\;y_{i}$—actual corn adulteration values; ${\hat{y}}_{i}$—predicted corn adulteration values; $\;{\overset{ˇ}{y}}_{i}$—mean of predicted corn adulteration values; ${\bar{y}}_{i}$—mean of actual corn adulteration values; σ—standard deviation of residual between actual and predicted corn adulteration values or SEC; S—the slope of the regression line.

**Table 3:** Model development results for adulteration quantification using partial least square regression (PLSR), multiple linear regression (MLR), and principal component regression (PCR) with the individual and combined sample set using pre-processed spectra (MAS + SNV + SG1d). The best model for each regression method is highlighted in bold. The RMSEC and RMSECV were expressed in % (*w*/*w*).

Model	Regression Method	LVs	R^2^_c_	R^2^_cv_	RMSEC	RMSECV	RPD_cv_
Individual wet model	**PLSR**	**5**	**0.93**	**0.89**	**3.85**	**4.80**	**2.99**
	MLR		0.87	0.87	5.44	5.20	2.76
	PCR	8	0.90	0.87	4.57	5.17	2.78
Individual dry model	**PLSR**	**6**	**0.92**	**0.89**	**3.93**	**4.87**	**2.94**
	MLR		0.84	0.84	6.00	5.75	2.49
	PCR	9	0.90	0.88	4.46	5.05	2.83
Global model	**PLSR**	**8**	**0.88**	**0.83**	**4.93**	**5.86**	**2.44**
	MLR		0.63	0.63	8.87	8.68	1.65
	PCR	9	0.72	0.69	7.52	8.02	1.78

**Table 4:** Prediction results for individual and combined prediction samples using the best individual and global PLSR models. The SEP, RMSEP, and bias were expressed in % (*w*/*w*).

Individual Wet PLSR Model	SEP	RMSEP	Bias	RPD_p_	RER_p_
Wet prediction samples	3.64	3.57	0.56	3.96	11.20
Dry prediction samples	11.43	45.59	−44.22	0.31	0.88
Combined prediction samples	24.23	32.33	−21.83	0.43	1.24
**Individual Dry PLSR Model**	**SEP**	**RMSEP**	**Bias**	**RPD_p_**	**RER_p_**
Wet prediction samples	9.61	50.96	50.10	0.28	0.78
Dry prediction samples	4.36	4.24	0.36	3.33	9.43
Combined prediction samples	26.31	36.16	25.23	0.38	1.11
**Global PLSR Model**	**SEP**	**RMSEP**	**Bias**	**RPD_p_**	**RER_p_**
Wet prediction samples	6.35	6.16	0.32	2.30	6.49
Dry prediction samples	5.48	5.38	0.94	2.63	7.43
Combined prediction samples	5.84	5.78	0.63	2.41	6.92

## Sample Availability

Samples of the compounds are not available from the authors.
